# Highly efficient method for isolation of total RNA from adipose tissue

**DOI:** 10.1186/1756-0500-6-472

**Published:** 2013-11-18

**Authors:** Susanna Cirera

**Affiliations:** 1 Department of Veterinary Clinical and Animal Sciences, Section for Animal Genetics, Bioinformatics and Breeding, Faculty of Health and Medical Sciences, University of Copenhagen, Frederiksberg, Denmark

**Keywords:** Adipose tissue, Total RNA isolation, RNA purity, RNA integrity

## Abstract

**Background:**

RNA extraction is a crucial step for monitoring gene expression. Poor RNA quality (including degradation and remaining impurities) can result in misleading results. Isolation of RNA from animal tissues with high lipid content can be challenging. Especially, it is not trivial to isolate high quality RNA with a reasonable yield from adipose tissue. The aim of this study was to provide an optimized protocol for isolating total RNA from adipose tissue. This was achieved by combining the advantages of the two routinely used methods, TRI Reagent® and miRNeasy.

**Findings:**

The miRNeasy method results in cleaner samples but more prone to degradation while the TRI Reagent® method results in samples contaminated with salts and solvents but more intact. The new protocol combines the best of both methods resulting in RNA of high quality and suitable for downstream experiments like RT-qPCR, microarrays and high-throughput sequencing.

**Conclusions:**

The current protocol for total RNA isolation from adipose tissue yields sufficient amount of high quality total RNA free of contaminants.

## Background

Adipose tissue has traditionally been viewed as a passive storage of energy in the body but has recently been recognized as a complex and highly active metabolic and endocrine organ [1]. As such, adipose tissues are of key importance for research in diabetes, metabolic syndrome and obesity which as a result of its epidemic proportions worldwide has become the focus of interest for many scientists.

Due to its high content in fatty acids and in some instances its low cell number, adipose tissue poses some challenges when high quality RNA needs to be isolated for downstream gene expression applications (i.e. arrays, high-throughput sequencing or RT-qPCR). Many routinely used protocols for isolation of RNA from adipose tissue result in RNA that is partially degraded and/or poor yield of RNA or the small RNAs are lost in the process. All these issues can cause misleading results and they are a big challenge specially when working with limited amount of tissue (i.e. human biopsies). Therefore there is a need for a simple, reliable and effective protocol to isolate high quality total RNA with sufficient yield from adipose tissues. Here an optimized and simple protocol which combines two widely used methods and allows rapid purification of high quality total RNA from adipose tissue is presented. Performance of the new protocol has been evaluated and compared with the standard methods for RNA purification.

## Methods

Porcine retroperitoneal adipose tissue, snap frozen in liquid nitrogen and stored at −80°C was used for the present study. Animal care, maintenance and slaughter have been conducted according to the Danish “Animal Welfare Act” (LBK 1343 of 04/12/2007).

Four samples (2 lean animals and 2 fat animals) were used for the TRI Reagent® (MRC Inc., US) and miRNeasy (Qiagen, Germany) methods. The 4 samples were the same for these 2 methods. For the combined protocol the number of samples was increased to 10 (5 lean animals and 5 fat animals). The phenotypic characterization of the animals as “lean” or “fat” is based on the body weight and on metabolic parameters [2].

**Figure 1:**
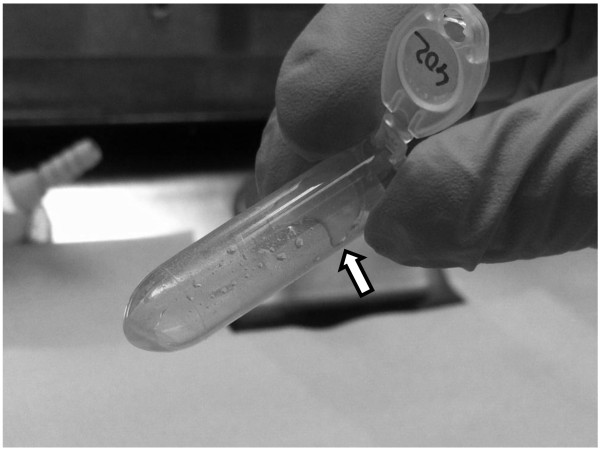
**Fat monolayer.** This fat monolayer appears after the first centrifugation step in all 3 protocols and it should be carefully avoided.

RNA isolation from adipose tissue was performed using TRI Reagent® method (MRC Inc., US) and miRNeasy method (Qiagen, Germany) and a combination of both (combined protocol). Around 100 mg of tissue were used to isolate total RNA with the three mentioned methods. Using TRI Reagent® and miRNeasy the exact protocols from the manufacturers were followed. For the combined protocol, the homogenization was done in 2 ml of TRI Reagent® buffer using a gentleMACS™ Octo Dissociator system (Milteny Biotec, GmbH, Germany) with M tubes (Milteny Biotec, GmbH, Germany) and the RNA_02 program recommended by the manufacturer. After homogenization the samples were incubated at room temperature for 5 minutes. Subsequently a centrifugation step at 12000 g at 4°C for 10 min was performed and the resulting fat monolayer (see Figure 1) was carefully avoided when pipetting the rest of the sample into a clean 1.5 ml tube. 400 μl of chloroform was then added to the sample and mixed by vortexing (thoroughly mixing is important for subsequent phase separation). After 3 minutes at room temperature the sample was centrifuged at 12000 g at 4°C for 30 min. After centrifugation, the sample separates into three phases with the RNA in the upper phase. The RNA phase was transferred to a new tube without disturbing the interphase. The volume of the sample was precisely measured and 1.5 volumes of 100% ethanol were added and the sample was mixed thoroughly by inverting the tube several times. The sample was then loaded on the miRNeasy spin column (Qiagen) and from this step the manufacturer’s protocol was followed. At the end the RNA was eluted in 30 μl of RNAse-free water.

**Figure 2:**
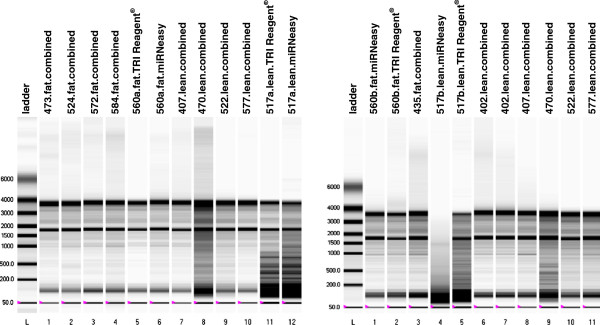
**Experion**^**TM **^**gel image.** The RQI corresponding values can be found in Table 1. Lane identity is given at the top of the gel image.

**Figure 3:**
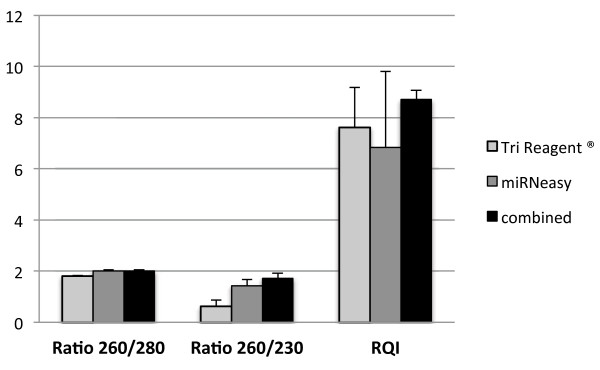
**Comparison of the 3 used methods for ratio 260/280, ratio 260/230 and RQI.** SEM is depicted by error bars.

Quantity and quality were assessed by Nanodrop ND-1000 spectrophotometer using OD_260_ for calculation of the concentration and the ratios 260/280 and 260/230 for assessing the purity of the samples. Integrity of the isolated RNA was inspected by electrophoresis in a 1.4% agarose gel and by measuring the RQI value on an Experion™ system (BioRad) using Eukaryote Total RNA StdSens kit (BioRad) (see Table 1 and Figure 2). RNA isolation from the three methods was evaluated and compared (see Table 1, Figure 2 and Figure 3). Student’s T-test was performed to compare the ratios 260/280 and 260/230 and the RQI between the 3 methods.

**Table 1:** Quantitative and qualitative measurements of the total RNA isolated with the 3 protocols

**Samples**	**Tissue amount**	**Protocol**	**Concentration**	**Ratio 260/280**	**Ratio 260/230**	**RQI**
560a.fat	100 mg	Tri Reagent®	125 ng/μl	1.83	0.85	9.5
560bfat	100 mg	Tri Reagent®	205 ng/μl	1.83	0.36	8.3
560a.fat	100 mg	miRNeasy	97 ng/μl	1.98	1.39	8.9
560b.fat	100 mg	miRNeasy	147 ng/μl	2.01	1.38	8.7
435.fat	100 mg	combined	92 ng/μl	1.94	1.46	8.3
473.fat	100 mg	combined	115 ng/μl	1.99	1.70	9.1
524.fat	100 mg	combined	147 ng/μl	2.02	1.45	8.9
572.fat	100 mg	combined	107 ng/μl	1.92	1.57	9.1
584.fat	100 mg	combined	107 ng/μl	1.97	1.74	8.6
517a.lean	100 mg	Tri Reagent®	109 ng/μl	1.79	0.45	6.4
517b.lean	100 mg	Tri Reagent®	89 ng/μl	1.79	0.81	6.3
517a.lean	100 mg	miRNeasy	128 ng/μl	2.02	1.78	7.2
517b.lean	100 mg	miRNeasy	109 ng/μl	2.05	1.17	2.5
402.lean	100 mg	combined	180 ng/μl	2.04	1.69	8.8
407.lean	100 mg	combined	125 ng/μl	2.00	1.90	9
470.lean	100 mg	combined	185 ng/μl	2.04	1.85	7.7
522.lean	100 mg	combined	144 ng/μl	2.05	1.88	8.6
577.lean	100 mg	combined	282 ng/μl	2.07	2.01	9.2

Tissue amount: starting amount of tissue used in each protocol; Ratios 260/280 and 260/230 measured by Nanodrop ND-1000; RQI measured by Experion™ machine; fat and lean samples: phenotype of the animals used based on weight and metabolic parameters described elsewhere [2].

## Findings and discussion

Due to the high lipid content and relatively low cell number of the adipose tissue, many methods for isolation of RNA from this tissue have been tested ([3-8], among others). Some of these methods are very time consuming and/or require high amount of starting tissue. Others do not report the purity as measured by microfluidics: I.e. Guan & Yang [4] and Duckett *et al.*[5] implemented a protocol similar to the combined method described in the present study. However, the authors did not test the integrity of the RNA with microfluidics. Verification of the RNA integrity by microfluidics (Experion or Bioanalyzer) is crucial in order to evaluate if the isolated RNA is of sufficient quality for downstream applications [9]. This shortcoming makes direct comparison of the methods difficult. Pena *et al.*[6] used a simplified version of the TRI Reagent® method but their RNA integrity results were considerably lower than the ones reported with our combined protocol. Recently, Pratt *et al.*[8] have developed a protocol (modified from Duckett *et al.*[5]) using the *miR*Vana miRNA Isolation kit and they report comparable yield and integrity results to our method. Nevertheless, the authors start the protocol with 1 gr of adipose tissue and the RNA integrity results are reported from only 2 samples.

In the present study a protocol for total RNA isolation from adipose tissue was developed based on widely used methods. The purity of the samples isolated with the new protocol was excellent: A 260/280 ratio of 2.00 ± 0.049 (1.81 ± 0.023 for TRI Reagent and 2.01 ± 0.03 for miRNeasy) and a 260/230 ratio of 1.73 ± 0.189 which was significantly higher (*P* < 0.05) than the other two (0.61 ± 0.25 for TRI Reagent and 1.43 ± 0.25 for miRNeasy). The RNA quality index (RQI) assessed by the Experion™ system resulted also in excellent values for the samples isolated with the new protocol, RQI = 8.7 ± 0.36 (7.62 ± 1.55 for TRI Reagent and 6.82 ± 2.98 for miRNeasy) which is quite unusual for adipose samples (see Table 1, Figure 2 and Figure 3).

The RNA yield was also higher for the combined protocol, 148.4 ± 56.25 (132 ± 50.84 for TRI Reagent and 120.25 ± 21.93 for miRNeasy).

The TRI Reagent® method showed problems with the purity of the isolated RNA. The ratio of 260/280 was lower than 1.8-2 indicating the presence of proteins. Furthermore, the 260/230 ratio, which should ideally be 1.8 or greater, was consistently very low (under 1) revealing remains of solvents (i.e. phenol/chloroform) or chaotropic salts (guanidine isothiocyanate) in the RNA solution (see Table 1). It is well known that all phenol-based methods have almost always little remaining of the organic solvents in the final isolated sample and that all these impurities remaining in the sample can compromise downstream applications.

The miRNeasy method resulted in RNA samples with 260/280 ratios around 2 and 260/230 ratios over 1. Nevertheless, the RNA yield for the samples originating from fat animals was 22-28% lower than with the TRI Reagent® method (see Table 1), even though we started both methods with the same amount of tissue and with exactly the same sample. Furthermore the integrity of the RNA, especially for the samples originated from lean animals was lower than with the TRI Reagent® method, indicating that the lipids carried over after the phase separation had more drastic effects during the process of the miRNeasy RNA isolation than with the TRI Reagent® method.

The homogenization step is very crucial for the quality of the final isolated RNA. Independently of the homogenization buffer used (TRI Reagent® or QIAzol) we experienced that if the disruption was performed using a mortar and pestle, the fatty acids were abundant in the sample after the homogenization and the fat monolayer was very evident after the initial centrifugation step (see Figure 1). In contrast, if the homogenization was performed using the gentleMACS™ machine most of the fat tended to stick to the walls of the M tubes used for homogenization and the monolayer, after the initial centrifugation step, was very small and easy to avoid and therefore the problem of fat carried over to the next steps was minimized. In the samples from lean animals, the fat did not stick so much to the plastic walls of the M tubes and the fat monolayer was more evident and difficult to avoid after the first centrifugation step. Therefore, the samples from lean animals performed worse than the samples originated from fat animals (lower 260/230 and 260/280 ratios, more degradation in the agarose gel and lower RQI, see Table 1 and Figure 2) in all the three protocols used in the present study but the effects were much less dramatic when using the new protocol.

In the step of phase separation, after addition of chloroform and 15 min. centrifugation at 12000 g and 4°C, the interphase was usually very diffuse and difficult to avoid. In order to make it more compact to avoid carrying it over when transferring the RNA upper phase, the centrifugation was extended from 15 minutes to 30 minutes.

When running the RNA samples in agarose gels or in the Experion™ system the samples from the miRNeasy protocol were slightly more degraded than the ones from the TRI Reagent® and the new protocols (see Table 1 and Figure 2).

## Conclusion

For total RNA isolation from adipose tissue, the miRNeasy method results in cleaner samples but more prone to degradation and the TRI Reagent® method results in samples contaminated with salts and solvents but more intact. The new protocol combines the best of both methods yielding sufficient amount of high quality total RNA from adipose tissue free of contaminants and suitable for downstream experiments like RT-qPCR, microarrays and high-throughput sequencing. At present, we use this protocol routinely in our laboratory starting from frozen adipose tissue and from isolated mature adipocytes with excellent results.

## Competing interests

The author declares not to have any competing interests.
